# Epineurectomy of extracranial facial nerve trunk for non-flaccid sequelae following Bell’s palsy: a single-arm trial

**DOI:** 10.1097/JS9.0000000000002080

**Published:** 2024-09-18

**Authors:** Hua Zhao, Xiaomin Cai, Zhongding Zhang, Tingting Ying, Yinda Tang, Haopeng Wang, Baimiao Wang, Shiting Li

**Affiliations:** Department of Neurosurgery, Xinhua Hospital Affiliated to Shanghai Jiao Tong University School of Medicine, Shanghai, China

**Keywords:** facial palsy, non-flaccid sequelae, synkinesis

## Abstract

**Background:**

Non-flaccid facial palsy sequelae manifest as sequelae following Bell’s palsy. Currently, there are no effective remedies for addressing this issue. In this study, the authors proposed a new surgical solution, epineurectomy of the extracranial facial nerve trunk, and assessed its safety and efficacy as a potential remedy.

**Methods:**

In this single-arm trial, adult patients with non-flaccid facial palsy sequelae were enrolled and subjected to epineurectomy of the extracranial facial nerve trunk. The primary efficacy endpoint was the Sunnybrook scores at months 12 postoperatively. The secondary endpoints included non-flaccid facial palsy sequelae symptom scores, such as facial tightness or facial stiffness, facial synkinesis, eye fissures narrowing or difficulty in opening the eyes, House-Brackmann grade scale, and Facial Disability Index.

**Results:**

A total of 22 patients were enrolled between July 2020 and January 2021. One patient was lost to follow-up. One year after surgery, the Sunnybrook score was 72.0 (63.0–75.0) at 12 months versus 68.0 (58.0–70.8) at baseline. The mean difference was −5.4 (−7.2 to −3.6). The scores of facial tightness or facial stiffness, synkinesis, eye fissures narrowing or difficulty in opening eyes were 0.0 (0.0–1.0), 1.0 (1.0–1.0), 1.0 (1.0–2.0) at 12 months versus 3.0 (1.3–3.0), 2.0 (1.0–2.8), 2.0 (2.0–3.0) at baseline, respectively. The median (IQR) values of the Facial Disability Index physical function were 92.0 (90.0–95.0) at months 12, and the mean difference (95% CI) was −32 (−38 to −26) compared to baseline. The mean difference (95% CI) in the Facial Disability Index social/well-being function between month 12 and baseline was −38 (−46 to −31).

**Conclusions:**

Epineurectomy of the extracranial facial nerve trunk can effectively and safely alleviate the sequelae of non-flaccid facial palsy.

## Introduction

Highlights

- Current treatments for non-flaccid facial palsy sequelae remain variable, including botulinum toxin injection, physical therapy, and surgical treatment (selective peripheral neurectomy and selective muscle resection), but their effectiveness is limited. Therefore, we established a new surgical procedure for epineurectomy of the extracranial trunk of the facial nerve to improve the chances of recovery from non-flaccid sequelae after Bell’s palsy.
- This study demonstrated that epineurectomy of the trunk of the facial nerve can treat non-flaccid post-facial palsy sequelae including synkinesis. And, this surgery had a certain percentage of advantages.

Bell’s palsy is traditionally defined as idiopathic acute unilateral peripheral facial palsy^1^ with an incidence of 20–30 per 100 000 people per year, accounting for 60–75% of all acute peripheral facial palsy cases^2^. Although Bell’s palsy is curable in most cases, 20–30% recover incompletely with residual facial palsy sequelae manifested by non-flaccid facial palsy^3^. The symptoms present as facial tightness or stiffness, synkinesis, narrowing of the eye fissure, or difficulty in opening the eyes. This type of sequela generally emerges between three and six months following the onset of Bell’s palsy^4^. These sequelae lead to functional limitations in the patient’s physical activities, including eating, drinking, smiling, and even disfigured facial expressions, eventually resulting in impairment of self-confidence and social isolation^5^.

The current treatment options for non-flaccid facial palsy sequelae are diverse and include botulinum toxin injections, physical therapy, and surgical interventions such as selective peripheral neurectomy and selective muscle resection. Despite their implementation, the efficacy of these treatments is reported to be limited^6^. Botulinum toxin injection results in drug resistance, and its efficacy decreases progressively^7^. Selective peripheral neurectomy and selective muscle resection not only have limited effectiveness but also cause more injuries and complications in patients^8,9^.

The pathogenesis of non-flaccid facial palsy sequelae is believed to involve ephaptic transmission between adjacent nerves or aberrant fiber regeneration. During the restoration process after Bell’s palsy, axons may regenerate incorrectly because of the disrupted integrity of the endoneurium tube, resulting in nerve fiber growth or sprouting into other endoneurium tubes, thus wrongly innervating facial muscles^5^. In a previous study, we performed ultrasonography of the extracranial facial nerve trunk (FNT) in patients with non-flaccid facial palsy sequelae and found significant changes in its lamellar structure, suggesting pathological changes in the extracranial FNT on the affected side^10^. The abnormal distribution of nerve fibers in the epineurium of the extracranial FNT was further verified by histological findings^10^. In-vivo animal experiments demonstrated that symptoms of non-flaccid facial palsy sequelae disappeared after the release and removal of the epineurium of extracranial FNT in a well-established Cavia porcellus model^10^.

Therefore, we established a new surgical procedure for epineurectomy of the extracranial trunk of the facial nerve to improve the chances of recovery from the non-flaccid sequelae of Bell’s palsy. In order to evaluate the efficacy and safety of this surgery, we performed this clinical study. The aim of this study is to assess the efficacy and safety of epineurectomy and perineurectomy of extracranial FNT for Bell’s palsy patients with spastic facial palsy sequelae via a clinical trial.

## Methods

### Trial design

This study was a single-arm clinical trial. The trial protocol was approved by the Institutional Review Board of Xinhua Hospital, Affiliated to Shanghai Jiao Tong University School of Medicine. The trial was performed in accordance with the principles of the Declaration of Helsinki and registered in the Chinese Clinical Trials Registry. Written informed consent was obtained from all participants prior to enrollment. All procedures were performed by a single surgical team led by a senior surgeon. All the listed authors made significant contributions and vouched for the completeness and accuracy of this report and its fidelity to the protocol. This study was in line with the STROCSS, Supplemental Digital Content 1, http://links.lww.com/JS9/D447 criteria^11^.

### Participants

The inclusion Criteria: 18–70 years old, no restrictions on sex, definitive history of Bell’s palsy, definitive symptoms of non-flaccid facial palsy sequelae, electrophysiological examination showing positive abnormal muscle response (AMR), accurate expression of subjective feelings, and willingness to participate in this research and sign an informed consent form before the study.

The major exclusion criteria were as follows: received treatments such as botulinum toxin and acupuncture within 3 months before enrollment; post-facial palsy sequelae caused by other secondary factors, including trauma and tumor, severe psychological or mental abnormalities, presence of severe systemic diseases; and participation in other clinical trials within 3 months prior to enrollment in this study.

### Interventions

**Figure 1:**
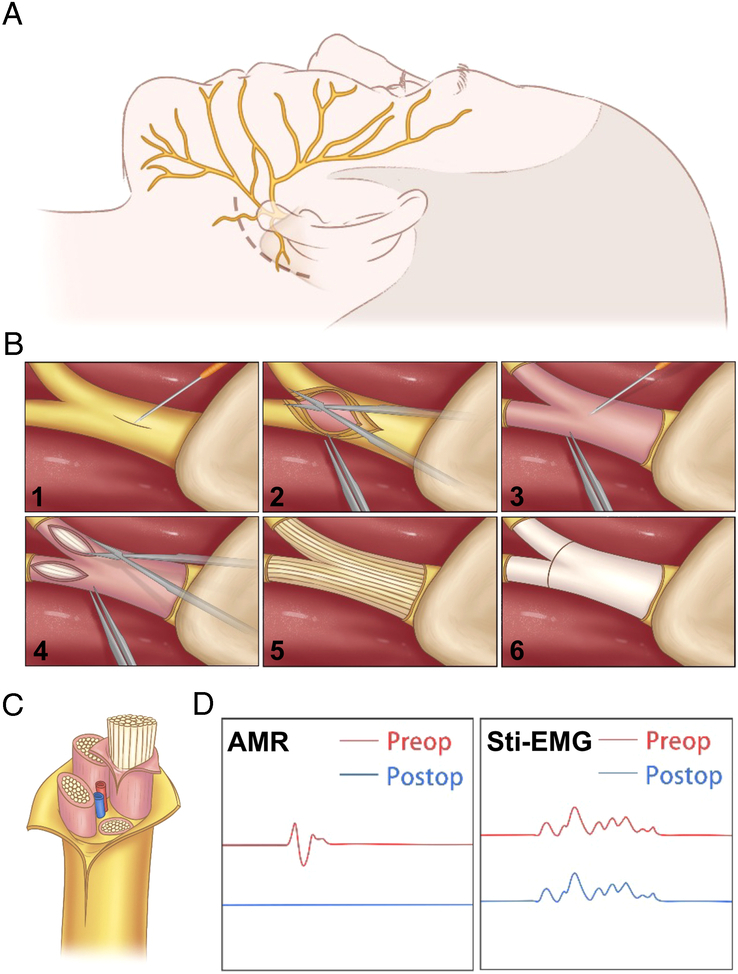
Schematic illustration of epineurectomy of the extracranial facial nerve trunk. (A) The patient was in the supine position, and the dotted line indicates the incision around the mastoid tip. (B) The epineurium of the facial nerve trunk was incised along the axis of the nerve using a custom-made sharp nerve neurolysis knife and released in a layer-by-layer manner using micro-scissors. Finally, the nerve was protected by using an artificial nerve sheath. (C) Schematic diagram of the cross-section of the main trunk of the facial nerve. Yellow and pink indicate the epineurium. (D) The AMR disappeared after the operation and the stimulus EMG (sti-EMG) did not change. AMR, abnormal muscle response.

The procedure for epineurectomy of the extracranial facial nerve trunk (FNT) is shown schematically in Fig. 1. All patients were placed in the lateral decubitus position and their heads were fixed in a frame, positioning the mastoid at the highest level under general endotracheal anesthesia^10^. Bipolar subdermal needle electrodes were inserted on the ill side. Electrical stimulation consisted of square-wave pulses (duration: 0.2 ms) adjusted to supramaximal strength with a frequency of 0.5 Hz. Electrical stimulation and electromyographic recordings were filtered through a 5 Hz–3 kHz bandpass (gain: 500 mV/division; analysis time: 50 ms). AMR recordings were obtained from the orbicularis oculi muscle by electrical stimulation of the marginal mandibular branch. A 4–6 cm long retroauricular curved incision was made around the mastoid tip. The sternocleidomastoid muscle was detached from the mastoid process to reveal the posterior aspect of the parotid gland, and an incision was made to explore the extracranial FNT. Blunt dissection was performed to separate the posterior belly of the digastric muscle from the parotid gland, thus increasing exposure of the posterior aspect of the parotid gland. Along the anterior margin of the digastric groove, the FNT was visualized beneath the posterior auricular artery on the deep side of the parotid gland. The final exposure length of the FNT was ~1–1.5 cm. If further exposure is required, the most proximate part of the FNT can be reached at the stylomastoid foramen level by drilling a portion of the mastoid tip. Before epineurectomy, the FNT was stimulated using a probe from the proximal end, and electromyography (EMG) images of the facial nerve branches (frontal, orbicularis oculi, orbicularis oris, and depressoranguli oris muscles) were obtained. The epineurium of the FNT was then separated and released layer-by-layer using micro-scissors. The procedure was performed under real-time AMR monitoring, and once the waveform disappeared, which usually occurs when the epineurium around the bifurcation region is released, the surgery was immediately stopped. The FNT was protected using an artificial nerve sheath (GI 5×30, Tech Thinkful, Beijing, China) to avoid postoperative adhesions. EMG of the facial nerve branches was re-recorded using a probe for comparison with that detected before epineurectomy. The latencies and amplitudes of the facial nerve branches showed no significant changes before and after epineurectomy, as evaluated by the monitoring physician. At last, the subcutaneous tissue and skin were sutured layer-by-layer, and the incision was disinfected and bandaged.

### Outcomes

The primary efficacy endpoint was the Sunnybrook scores at months 12 changed from baseline, as assessed by two independent investigators.

The secondary endpoints were divided into five parts. (1) The Sunnybrook sub-items of synkinesis and voluntary movement—resting symmetry were used to evaluate facial synkinesis and facial flaccid symptoms, respectively. Non-flaccid facial palsy sequelae were assessed using a questionnaire consisting of the following three domains (score of 0–3 for each domain, with higher scores indicating more severe disability): facial tightness or facial stiffness, facial synkinesis, and eye fissure narrowing or difficulty opening the eyes.

Facial nerve function was assessed using the House-Brackmann (HB) grade scale. The physical or social/well-being functions of the patients were evaluated using the Facial Disability Index (FDI). Follow-up was conducted at 1 week and at 3, 6, and 12 months after surgery.

### Statistical analysis

The trial sample size was calculated to ensure 80% power to detect a 5-point (SD=5) increase in the Sunnybrook score and any adverse events or other safety outcomes that had an expected incidence of at least 8%. According to retrospective data, the median Sunnybrook score subitem synkinesis was 10, and we expected that surgery would lead to a half reduction of synkinesis, and thus a 5-point increase in Sunnybrook score.

The primary outcome analysis was performed using the full analysis set (FAS). For continuous outcomes, the median (95% CI) of the paired difference between baseline and one year after surgery was estimated and tested using the Wilcoxon matched-pair signed-rank test. Categorical outcomes were analyzed using the McNemar’s test. No multiplicity adjustment was performed for secondary endpoints. Sensitivity analysis was conducted for each protocol set (PP) for the primary outcome. AEs were assessed using a safety set (SS). Multiple imputations were used to address missing data. All statistical analyses were performed using R software (version 4.2). The independent variables were analyzed using multiple factor logistic regression. Statistical significance was set at *P* less than 0.05 (two-sided) were considered statistically significant.

## Results

### Patients

Twenty-six patients were screened between July 2020 and January 2021, and 22 were eligible for inclusion and were enrolled. The remaining four patients were not enrolled for the following reasons: extremely severe systemic diseases (one patient), declined to sign informed consent (two patients), and facial palsy sequelae caused by the tumor (one patient). A detailed flow diagram of patient enrollment and follow-up is shown in Supplementary Figure 1, Supplemental Digital Content 1, http://links.lww.com/JS9/D448.

The median [IQR] age of the patients was 42 [31.8, 53.0] years, and most were female (14, 64%). Facial palsy predominantly occurred on the left side (*n* = 14, 64%). The duration of facial palsy was defined as the interval between the start of the facial palsy and the time of entry into the trial. Correspondingly, the time from the appearance of post-facial palsy synkinesis to surgery was the duration of sequelae. The median [IQR] duration of facial palsy or sequelae was 24 [10.5, 63.0] or 12 [4.3, 60.0] months, respectively. The degree of facial palsy in House-Brackmann (HB) scores at baseline was HB grade II (9 patients) and HB grade III (13 patients). Notably, nine patients received one or more BTX injections without significant benefits before entering the clinical trial (Table 1). At baseline, all patients suffered from non-flaccid facial palsy sequelae, with different degrees of symptoms, including facial tightness or facial stiffness, facial synkinesis, narrowing of eye fissures, or difficulty in opening their eyes.

**Table 1:** Characteristics of the patients at baseline.

Characteristics	Overall (*n*=22)
AGE (median [IQR])	42.00 [31.8, 53.0]
Female sex, *n* (%)	14 (64)
Side = Right, *n* (%)	8 (36)
Duration of facial palsy (M, months), (median [IQR])	24.00 [10.5, 63.0]
Time from appearance of post-facial palsy synkinesis to surgery (M, months), (median [IQR])	12.00 [4.3, 60.0]
Abnormal muscle response, *n* (%)	22 (100)
Botulinum Toxin injection, *n* (%)	9 (40.9)
Sunnybrook scores	
Synkinesis^a^	10.0 (7.3–12.0)
Voluntary movement—resting symmetry	74.5 (70.0–82.0)
Voluntary movement—resting symmetry—synkinesis	68.0 (58.0–70.8)
Non-flaccid sequelae^b^	
Facial tightness or facial stiffness	3.0 (1.3–3.0)
Synkinesis	2.0 (1.0–2.8)
Eye fissures narrowing or difficulty in opening eyes	2.0 (2.0–3.0)
House-Brackmann Facial Grading System, *n* (%)^c^	
II	9 (40.9)
III	13 (59.1)
ENoG amplitude (mV), (median [IQR])^d^	1.7 (1.2–2.5)

ENoG, electroneurography; IQR, interquartile range.

a Sunnybrook sub-item score was calculated separately. As for voluntary movement—resting symmetry, higher score represents better facial nerve function. As for Synkinesis, higher score means the more serious degree.

b The score of three kinds of non-flaccid sequelae symptoms was presented, with 0–3. (0 represents normal function; 3 represents the worst possible dysfunction.).

c House-Brackmann (HB) Facial Grading System is graded by the static and dynamic condition of the face and has six grades, with grade I–VI (completely normal to completely immobile).

^d^ The digits indicate wave amplitude of ENoG of the affected side.

Data are *n* (%) or median (IQR), unless otherwise specified.

### Primary outcome

**Figure 2:**
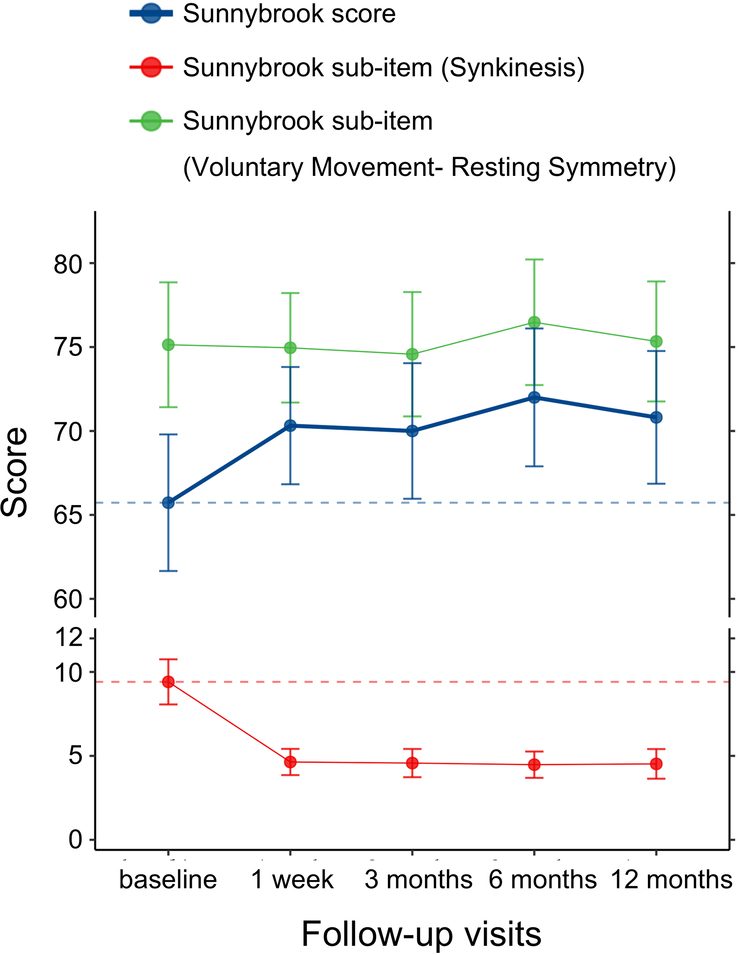
Sunnybrook score over the 12-month study period.

The median (IQR) values for the total Sunnybrook score were 72.0 (63.0–75.0) at 12 months versus 68.0 (58.0–70.8) at baseline, indicating a remarkable improvement at 12 months after surgery (difference, −5.4; 95% CI, −7.2 to −3.6; *P*<0.001) (Table 2). As shown in Fig. 2, the patients’ Sunnybrook scores improved most markedly at 1 week postoperatively, and remained stable until 12 months.

**Table 2:** Primary and secondary outcomes at month 12.

Outcomes	Overall (*n* = 21)	Mean difference (95 CI)	*P*
Primary outcome			
Sunnybrook scores^a^	72.0 (63.0–75.0)	−5.4 (−7.2 to −3.6)	<0.001
Secondary outcomes			
Sunnybrook sub-items^b^			
Synkinesis	5.0 (4.0–5.0)	−5.2 (−3.9 to −6.5)	<0.001
Voluntary movement—resting symmetry	74.0 (70.0–82.0)	−0.19 (−1.1 to 0.72)	0.67
Non-flaccid sequelae^c^			
Facial tightness or facial stiffness	0.0 (0.0–1.0)	−1.7 (−1.2 to −2.2)	<0.001
Synkinesis	1.0 (1.0–1.0)	−1.2 (−0.86 to −1.6)	<0.001
Eye fissures narrowing or difficulty in opening eyes	1.0 (1.0–2.0)	−0.43 (−0.20 to −0.66)	<0.001
Facial Disability Index physical function^d^	92.0 (90.0–95.0)	−32 (−38 to −26)	<0.001
Facial Disability Index social/well-being function^d^	90.0 (88.0–96.0)	−38 (−46 to −31)	<0.001

a Sunnybrook scores is used to subjectively assess the face, out of 100, the higher the score the better the facial nerve function.

b Sunnybrook sub-item score was calculated separately. As for voluntary movement—resting symmetry, higher score represents better facial nerve function. As for Synkinesis, higher score means the more serious degree.

c The score of three kinds of non-flaccid sequelae symptoms was presented, with 0–3. (0 represents normal function; 3 represents the worst possible dysfunction.).

^d^ Facial Disability Index (FDI) is used to assess the physical and social/well-being function, with 0–100 (worst to best).

### Secondary outcomes

The Sunnybrook score consists of three parts including voluntary movement, resting symmetry, and synkinesis. Next, we attempted to determine which part of the Sunnybrook score changed most noticeably after surgery. The results showed a remarkable decrease in the Sunnybrook subitem (synkinesis) at months 12. The Sunnybrook sub-item (voluntary movement—resting symmetry) did not change after surgery, indicating that the reason for the improvement in the total Sunnybrook score was a decrease in the synkinesis score (Fig. 2).

**Figure 3:**
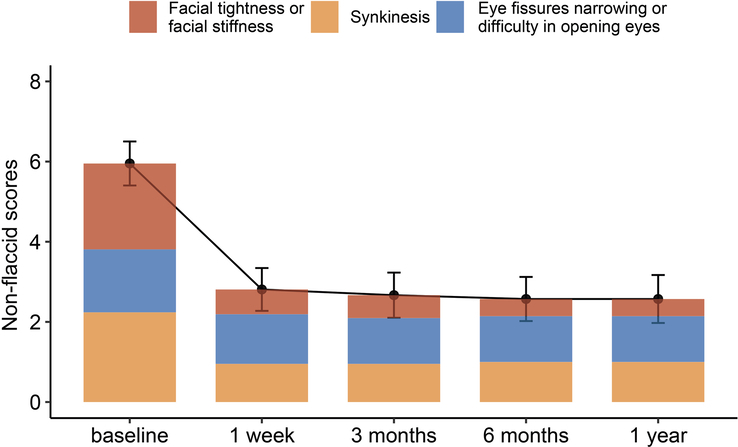
Non-flaccid facial palsy sequelae score over the 12-month study period. Orange, yellow, and blue represent facial tightness or facial stiffness, synkinesis, and eye fissure narrowing or difficulty in opening the eyes, respectively.

The median (IQR) values for facial tightness or stiffness were 0.0 (0.0–1.0) at month 12 versus 3.0 (1.3–3.0) at baseline, suggesting a marked improvement at months 12 after surgery (difference, 1.7; 95% CI, 1.2–2.2; *P*<0.001) (Table 2). The mean difference (95% CI) in synkinesis between months 12 and baseline was −1.2 (−0.86 to −1.6), suggesting a good recovery of synkinesis. The patients’ facial tightness, stiffness, and synkinesis improved most markedly at 1 week postoperatively and remained stable for 12 months (Fig. 3). However, non-flaccid facial palsy sequelae, such as eye fissure narrowing or difficulty opening the eyes, improved only subtlety (Fig. 3).

Improvement in patients’ physical function or social/well-being was measured using the Facial Disability Index (FDI). The median (IQR) values of FDI physical function were 92.0 (90.0–95.0) at months 12, and the mean difference (95% CI) was −32 (−38 to −26) compared with baseline. The mean difference (95% CI) in FDI social/well-being function between month 12 and baseline was −38 (−46 to −31), revealing that patients’ facial disability improved significantly (Table 2).

**Figure 4:**
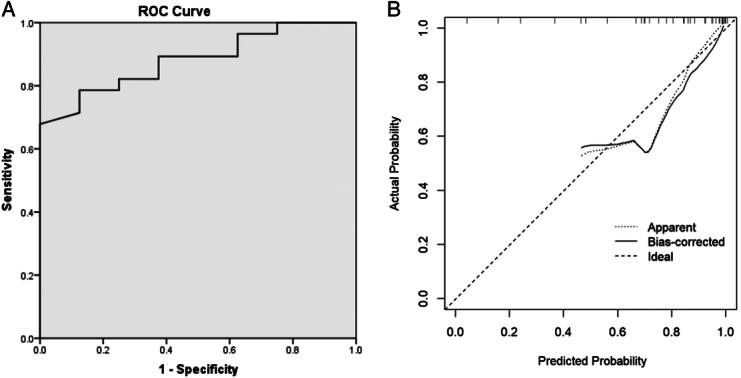
Logistic regression modeling was performed on the two independent variables (age and Sunnybrook scores). (A) The area under the ROC curve was 0.882. (B) The calibration curve indicates good model performance. ROC, receiver operating characteristic.

Logistic regression analysis showed that the prognosis of this disease with age and Sunnybrook score was statistically significant (*P*<0.05). These two factors are risk factors that affect surgical prognosis. Logistic regression modeling was performed on the two independent variables, and the area under the ROC curve was 0.882. The calibration curve indicates good model performance (Fig. 4).

### Safety

Ten patients showed mild facial palsy aggravation immediately after the operation (HB increased by 1 grade), but all patients recovered to baseline level at months 6. Adverse events associated with this surgery [as determined by the principal investigator (the last author)] included incision numbness in 17 patients, incision pain in 18 patients, and fever in three patients within 1 week after surgery. Fever and incision pain completely resolved at 3 and 6 months, respectively. Only two patients still complained of incision numbness at months 12. We observed no other adverse events that severely affected patients’ daily lives (Table 3).

**Table 3:** Adverse Events.

Complications related to treatment—no. events^a^					
Events	Baseline	1 week	3 months	6 months	1 year
No. patients whose HB has risen by 1 grade	0	10	4	0	0
Fever	0	3	0	0	0
Bleeding	0	0	0	0	0
Incision infection	0	0	0	0	0
Incision numbness	0	17	10	5	2
Incision pain	0	18	2	0	0
Hearing	0	0	0	0	0

a The principal investigator determined whether a complication was related to the study treatment. A complication on either side of the body is included.

HB, House-Brackmann.

## Discussion

Several surgical procedures, including selective myectomy and neurectomy, have been proposed. Unfortunately, these approaches have proven to be insufficient and often unpersuasive. In 1986, DOBIE and FISCH applied selective neurotomy to treat patients with non-flaccid facial palsy sequelae^12^. Short-term follow-up results showed satisfactory efficacy; however, all patients experienced narrowing of the affected eye fissure 2–3 years after surgery. Subsequently, some scholars improved the procedure and proposed a two-step highly selective neurotomy (2HSN) for the treatment of patients with non-flaccid facial palsy sequelae; however, long-term follow-up results showed recurrence of non-flaccid facial palsy sequelae^13^. Masseter nerve facial nerve anastomosis is commonly used to improve synkinesis; however, this procedure is highly invasive, involves multiple steps, and requires high microsurgical anastomosis skills from surgeons. The postoperative efficacy lacks long-term follow-up results. These factors have limited the promotion and application of this technique^14^.

The present single-center study showed that epineurectomy for extracranial FNT led to a significant improvement in non-flaccid sequelae symptoms after Bell’s palsy. Notably, the proposed surgical intervention presented more promising and long-lasting results than botulinum toxin (BTX) injection, which has been reported to last for only 2–3 months after each injection^15^. BTX injection has been a well-established benefit in the control of post-palsy facial synkinesis in the literatures^16–18^. However, its application has many disadvantages such as multiple injection sites, repeated injections, anaphylaxis, blepharoptosis, diplopia, and acceleration of the facial palsy^19^. More importantly, the effect of BTX continues to decrease following repeated injections, which destroys the self-confidence and patience of patients^19^. Adverse events associated with this surgery included incision numbness and pain, and only two patients complained of incision numbness at month 12, indicating the superior safety of the surgery.

Our findings demonstrated that among the three types of non-flaccid facial palsy sequelae, facial stiffness and tightness improved the most significantly, followed by synkinesis. We speculate that the reason for this phenomenon is that some aberrant proliferating nerve fibers after Bell’s palsy were removed during this procedure, resulting in improved facial stiffness and tightness. Post-facial palsy synkinesis also decreases at different levels in most patients with Bell’s palsy. This reminded us to meticulously reconsider which types of patients with facial palsy sequelae fit the surgery that we proposed better. According to the results of our study, patients with severe non-flaccid symptoms such as facial stiffness and tightness could benefit more from this operation.

Before surgery, electrophysiological examination was a useful tool for identifying Bell’s palsy patients with non-flaccid facial palsy sequelae. Patients with AMR waveforms were capable of being enrolled in the study. The importance of intraoperative electrophysiological monitoring should also be emphasized. Continuous electrophysiological monitoring is essential during the entire surgical course. To the best of our knowledge, AMR waveforms of post-facial palsy synkinesis are similar to those of hemifacial spasms^20^. However, the pathophysiological mechanism of facial synkinesis-related AMR is not vascular compression but aberrant regeneration of nerve fibers^10,21,22^. When the AMR waveform was stably detected after full metabolism of muscle relaxants, we started epineurectomy of extracranial FNT under real-time AMR monitoring. In addition, we regarded the disappearance of the AMR waveform as a sign of termination of surgery. During the entire course of surgery, the role of the AMR waveform was as important as that of hemifacial spasm. This helped us decrease the possibility of extra FNT injuries caused by surgical manipulation. Hence, we recommend the application of AMR monitoring in epineurectomy of extracranial FNT.

No permanent aggravation of fascial palsy was observed in any patient in the present study. Only 10 patients had temporary deterioration of facial palsy postoperatively but recovered absolutely at the 1-year follow-up. The possible reasons for this are as follows: (1) Surgical intervention might cause some damage to miniature nerve fibers owing to the limitations of the microscope during epineurectomy of extracranial FNT. (2) Inflammation and swelling in the surgical area might compress the facial nerve, although we have already utilized an artificial nerve sheath to wrap the nerve with the expectation of avoiding the abovementioned situation. The duration of facial palsy was defined as the interval between facial palsy and nerve reconstruction^23^. Previous studies^22,23^ have demonstrated that prolonged duration of facial palsy might cause adverse changes in the axonal microenvironment within the facial nerve, including degeneration of Schwann cell tubules within the distal nerve stump and motor endplates, eventually leading to atrophy of the distal target muscles^23^. Su *et al* ^23^. proposed that early facial nerve repair is essential to improve functional facial recovery. This prompted us to investigate whether the duration of facial palsy could affect non-flaccid post-facial palsy sequelae symptom improvement after surgery. However, no definite correlation was observed between the duration and post-facial palsy sequelae in this study. Some possible reasons may include the following: the sample size was small, and only 22 patients were enrolled, with the potential to cause bias; some patients were obliged to delay their time to visit the hospital because of their own affairs, thus remarkably changing the time course of the disease.

Taken together, this study demonstrates that epineurectomy of extracranial FNT can treat non-flaccid post-facial palsy sequelae including synkinesis. And, this surgery had a certain percentage of advantages. (1) This procedure is a minimally invasive operation; only a 4–6 cm long retroauricular curved incision was made around the mastoid tip, and there were no complications, such as subcutaneous infection, parotid gland fistula, middle ear mastoid effusion, or external auditory canal injury after the operation. The incision had healed well. Compared with the surgical methods reported in the literature, our study provides a more minimally invasive treatment approach. (2) The surgical procedure was standardized owing to the relatively constant anatomical position of the extracranial FNT, indicating its huge potential for further clinical popularization. In addition, we have provided an effective method for treating this disease and electrophysiological evidence for its diagnosis and treatment.

### Limitations

However, the present study has some limitations. First and foremost, it is essential to acknowledge the potential limitations of this study, which may have arisen due to the small number of cases and the fact that only one hospital participated in the recruitment of patients. As a result, the clinical trial became a single-center study. Therefore, to avoid these problems, larger multicenter studies are required to confirm our preliminary results. Second, the proposed surgical modality was not compared with other surgical or nonsurgical modalities such as physical therapy, biofeedback, and BTX. However, their effectiveness has been limited. Therefore, we did not include a control group in the present study. Therefore, large-scale randomized controlled clinical trials are required. Third, advances in neurolysis knives and microscopes may further improve the benefits for patients with non-flaccid post-facial palsy sequelae brought by this surgery.

## Conclusions

Epineurectomy of the extracranial facial nerve trunk can effectively and safely alleviate non-flaccid facial palsy sequelae.

## Ethical approval

This research study is approved by Xinhua hospital affiliated to Shanghai Jiaotong University school of medicine. Number:XHEC-C-2020-028.

## Consent

The written consent is available for review.

## Source of funding

This project was supported by Shanghai Science and Technology Commission (grant nos. YDZX20233100003001 and 21Y21900500 to S.L.), National Natural Science Foundation of China (grant nos. 82171360 to S.L.) and the Foundation for Interdisciplinary Research of Shanghai Jiao Tong University (grant no. YG2022QN042 to H.Z.).

## Author contribution

H.Z.: writing—original draft, funding acquisition. X.C.: data curation, writing—original draft, funding acquisition. T.Y.: methodology, investigation. Z.Z.: statistic analysis. Y.T.: resources, supervision. H.W.: software, validation. B.W.: visualization, writing—review and editing. S.L.：conceptualization, funding acquisition, supervision, writing—review and editing.

## Conflicts of interest disclosure

The authors declares no conflicts of interest.

## Research registration unique identifying number (UIN)

This research was registered at the Chinese Clinical Trials Registry (ChiCTR2000033545; June 5, 2020).

## Guarantor

Shiting Li.

## Data availability statement

Not applicable.

## Provenance and peer review

Not commissioned, externally peer-reviewed.

## Supplementary Material
