# Supplementary figures and images for: Epineurectomy of extracranial facial nerve trunk for non-flaccid sequelae following Bell’s palsy: a single-arm trial
Source: Int J Surg. 2024 Sep 18;111(1):536–42. doi: 10.1097/JS9.0000000000002080 (PMC11745721; doi:10.1097/JS9.0000000000002080)

**Figure S1.** CONSORT diagram.

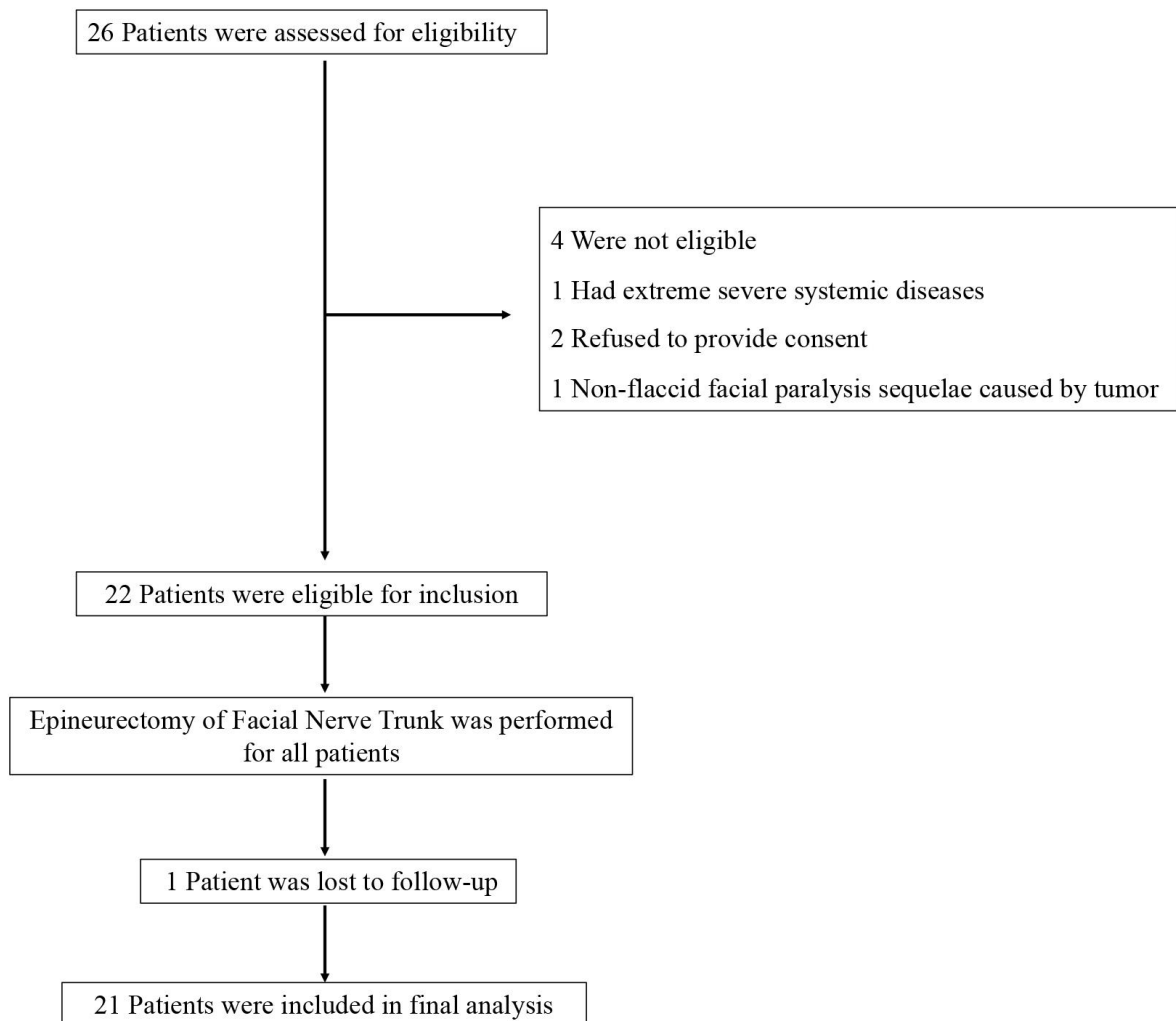

Supplement: Supplementary file 2 [file js9-111-0536-s002.pdf]
